# Precision Medicine Needs to Think Outside the Box

**DOI:** 10.3389/fgene.2022.795992

**Published:** 2022-04-26

**Authors:** Daphne O. Martschenko, Jennifer L. Young

**Affiliations:** ^1^ Center for Biomedical Ethics, School of Medicine, Stanford University, Stanford, CA, United States; ^2^ Center for Genetic Medicine, Feinberg School of Medicine, Northwestern University, Chicago, IL, United States

**Keywords:** precision medicine, GWAS, genome-wide association study, multiracial, admixed, equity, mixed ancestry

## Abstract

Precision medicine offers a precious opportunity to change clinical practice and disrupt medicine’s reliance on crude racial, ethnic, or ancestral categories by focusing on an individual’s unique genetic, environmental, and lifestyle characteristics. However, precision medicine and the genomic studies that are its cornerstone have thus far failed to account for human diversity. This failure is made clearer when looking at individuals who encapsulate a mosaic of different genetic ancestries and do not fit neatly into existing population labels. This piece argues that precision medicine continues to rely on the same forms of crude categorization it seeks to unsettle. Until the scientific community creates inclusive solutions for individuals who fall outside or between our existing population labels, precision medicine will continue to fall short in its aims.

## Introduction

An increasing number of individuals are defying the crude systems of racial, ethnic, and ancestral categorization used in medicine and society. For instance, over the past decade, the number of Americans who self-identify as multiracial has more than doubled (https://www.census.gov/library/stories/2021/08/improved-race-ethnicity-measures-reveal-united-states-population-much-more-multiracial.html) and increased globalization and population migration have resulted in greater genetic admixture—defined as the recent combination of two or more genetic ancestries. (Korunes and Goldberg, 2021). For those of us who do not fit neatly into existing racial, ethnic, or ancestral population labels, the problematic practice of categorizing people into discrete groups can be especially exclusionary. Precision medicine is one area in which such individuals are being left behind. In this Commentary, we argue that realizing the aims of precision medicine requires the medical genomics community to comprehensively study and analyze data from those who cannot be classified into existing population labels.

### Precision Medicine

Precision medicine examines how an individual’s unique genetic, environmental, and lifestyle characteristics come together to inform health. Instead of one-size-fits-all approaches to medical decisions, interventions, and treatments, precision medicine focuses on customization to the individual. Central to enabling such customization is medical genomics research–a heavily funded research priority for precision medicine (https://www.genome.gov/news/news-release/NHGRI-awards-73million-to-continue-building-Clinical-Genome-Resource-ClinGen). Researchers in medical genomics use genome-wide association studies (GWAS) to identify fine-grained differences in the DNA sequences of related and unrelated individuals. Aggregating the small effects of thousands of genetic variants identified through GWAS, polygenic scores (PGS) are used to estimate a person’s likelihood of exhibiting a particular phenotype (e.g., cardiovascular disease). Recent efforts in precision medicine have focused on how PGS might be used in combination with environmental risk scores (ERS) to screen individuals for diseases such as cancer.

The advent of precision medicine offers a precious opportunity to move beyond mutually exclusive categories such as race (Raut et al., 2021; Bonham et al., 2016) and account for human genetic diversity among individuals within and between populations. (Lewontin et al., 1972). GWAS and PGS could enable clinicians to make more accurate diagnoses and tailor treatments using an individual’s genome instead of self-identified or inferred race or ethnicity. However, while precision medicine carries the promise of improving clinical care, preventing and treating disease, and rejecting the use of race-based corrections in medicine, (Ashley, 2015; Cerdeña et al., 2020), it has thus far failed to deliver. Such failures are made clearer when examining how medical genomics handles admixed individuals who encapsulate more than one genetic ancestry.

### The Limitations of Precision Medicine for Admixed Individuals

Despite the rapidly decreasing costs associated with conducting GWAS, the overwhelming majority of genomic studies use samples from European genetic ancestries (https://gwasdiversitymonitor.com/); this restricts the potential benefits of genomics research on health to a narrow subset of the global population while also introducing sampling bias. (Popejoy and Fullerton, 2016). The challenges of population stratification, coupled with Euro-centric biases in genomic databases, mean that PGS derived from GWAS have systematically lower predictive performance when applied to understudied populations. As a result, the disease risk of non-European populations, including admixed populations, are either under- or over-estimated using existing PGS. (Martin et al., 2017). Any benefits afforded by PGS are less likely to accrue among people of non-European ancestry and more likely to exacerbate health disparities in disease treatment. (Martin et al., 2019).

In an effort to increase and diversify the sampling of participants, we must build databases that better reflect the global population, and widen the applicability of precision medicine research. Initiatives such as the NIH-funded *All of Us* Research Program are emerging (https://allofus.nih.gov/) to respond to this unmet need. However, initiatives such as these will never realize the full benefits of precision medicine unless explicit attention is devoted to finding ways to study admixed individuals in medical genomics research; this includes both existing admixed populations (e.g., Hispanic or Latin American) and recently admixed individuals who fall outside of already-defined admixed population categories.

Although genetics researchers are beginning to conduct studies with samples from diverse populations that encapsulate more than one genetic ancestry (e.g., self-identified African American or Hispanic/Latin American), (Wojcik et al., 2019; Gopalan et al., 2021), the vast majority of studies continue to deprioritize and discard admixed samples, citing inadequate sample sizes and technical complexities. (Peterson et al., 2019; Ben- Eghan et al., 2020).

These issues are further exacerbated for recently admixed individuals. First and second-generation admixed individuals are often grouped into monolithic categories such as ‘Other admixed ancestry,’ (Morales et al., 2018) ‘Other and other admixed,’ or ‘Multiple’ (https://www.ebi.ac.uk/gwas/docs/ancestry-data). Aggregating individuals into these categories may help to increase statistical power, but it denies researchers opportunities to examine relationships between the unique sociocultural factors and genetic characteristics that come together to shape an individual’s health and well-being.

Current genomic methods are especially insufficient for analyzing data from first and second generation admixed individuals. Continental ancestry categories (e.g., European, African) are the most common type of group label in genomics research. (Panofsky and Bliss, 2017; Lewis et al., 2021). The overreliance on continental ancestry categories not only encourages dangerous slippage between genetic ancestry and race, (Panofsky and Bliss, 2017), it disincentivizes researchers from finding ways to include those who fall outside a broad continental grouping. For instance, an individual who is a recent combination of Greater Middle Eastern genetic ancestry and South East Asian genetic ancestry is likely to be categorized as ‘Other and other admixed’ and will be discarded from genomic analyses because they cannot be assigned to a distinct regional population grouping.

The current limitations of medical genomics raise important scientific and ethical considerations regarding missed scientific opportunities, underrepresentation in research, and participants’ efforts to contribute to science. It is ethically problematic to continue inequitable resource allocations that drive underrepresentation in genomic studies, (Fatumo et al., 2022), just as it is ethically problematic to recruit participants for research and then discard their contributions from analyses. The consequence of such practices for precision medicine is that many do not currently stand to benefit from research into pharmacogenetics or disease risk prediction and will continue to be left behind even as the field outwardly seeks to diversify biobanks.

### Possible Solutions

To address these issues, precision medicine must first recognize, incorporate, and amplify the work of researchers who are already grappling with issues of diversity and equity in clinical and healthcare contexts in and outside of genetics. (Panofsky and Bliss, 2017; Lewis et al., 2021). This means expanding the range of voices given decision-making capacities and committing to an ethos of diversity in research and the workplace. (McFarling, 2021)^,^ (Thomas et al., 2021) Researchers must also prioritize community-engaged efforts that focus on building dynamic two-way partnerships instead of transactional exchanges for which data collection is an endpoint. Implementing more inclusive approaches to how precision medicine is carried out will introduce new perspectives and ways of thinking that can help to improve our current methods of analysis in genomics to account for admixed individuals.

In support of improving health outcomes and enhancing disease prevention and treatment, precision medicine should also consider whether existing systems of classification, methodological approaches, and research priorities are appropriate. We join others in cautioning against our default use of continental ancestral groupings in genetics. (Lewis et al., 2021). Although admixed individuals, who are considered a mixture of broad continental groups, may be used to compound population labeling, (Lewis et al., 2021), we believe that admixed individuals such as ourselves offer a chance to escape from it. The limited framework for attaining diversity in genomics have negative consequences for those of us who do not fit into a box. Therefore a critical and reflexive audit of how precision medicine research is conducted, who it benefits, and the changes required, calls for additional specific attention to those who cannot be classified using our current population schema. Studying rather than ignoring recently admixed populations is not only a scientific and ethical imperative, it will provide opportunities to develop novel methods and analytic techniques that resist continental ancestry groups and help realize the full potential of precision medicine for all. (Peterson et al., 2019).

Finally, precision medicine initiatives must prioritize investigations of the social context and the role of social and environmental factors including structural racism in shaping human health. If we want precision medicine to benefit all and not just some, the research enterprise needs to understand the systems-level factors that contribute to health disparities. (Newman, 2021). Individuals who defy the crude systems of racial, ethnic, and ancestral categorization used in medicine and society carry unique lived experiences that cannot be captured by genetics alone. These experiences are shaped by social contexts and hold potentially important health implications. Understanding the multitude of ways that individuals who do not fit into a box experience health is critical to offering genuinely customizable healthcare.

## Conclusion

Precision medicine is failing those who do not fit neatly within our crude systems of categorization—whether they be racial, ethnic, *or* genetic ancestral. The limitations of precision medicine for recently admixed individuals who cannot be described using existing population labels illustrate this. Precision medicine will not dismantle our reliance on reductionist categorizations by using the very tools that require them. And, it will not improve health outcomes with biased genomic databases that leave out large swaths of the global population and distract from the social structures and systems that contribute to health. Until we recognize the limitations of our approach to precision medicine and seriously grapple with who it leaves out, we cannot rely on it to systematically improve how we prevent and treat disease.

## Data Availability

The original contributions presented in the study are included in the article/Supplementary Material, further inquiries can be directed to the corresponding author.
